# Smart home technology to support older people's quality of life: A longitudinal pilot study

**DOI:** 10.1111/opn.12489

**Published:** 2022-07-04

**Authors:** Christina Aggar, Golam Sorwar, Carolyn Seton, Olivia Penman, Anastasia Ward

**Affiliations:** ^1^ Faculty of Health Southern Cross University Bilinga QLD Australia; ^2^ Feros Care Tweed Heads NSW Australia

**Keywords:** automation, caregivers, independent living, older people, quality of life, smart home

## Abstract

**Aim:**

This pilot study aimed to explore the impact of Smart Home technology to support older people’s quality of life, particularly for those who live alone.

**Background:**

There has been an increased interest in using innovative technologies and artificial intelligence to enable Smart Home technology to support older people to age independently in their own homes.

**Methods:**

This study used a pre‐and post‐test design. The seven item Personal Wellbeing Index was used to measure participants’ subjective quality of life across seven quality of life domains. Participants (*n* = 60) aged between 68 and 90 years (*M* = 80.10, SD = 5.56) completed a 12‐week personalised Smart Home technology program.

**Results:**

Approximately half of the participants lived alone (48.3%). Participants’ quality of life significantly increased (*p* = 0.010) after Smart Home use. Two domains, “achieving in life” (*p* = 0.026) and “future security” (*p* = 0.004), were also significantly improved after participating in the Smart Home technology program. Improvements in quality of life did not vary as a function of living arrangement (all *p*s > .152, all $\eta_{p}^{2}$ > .00).

**Conclusion:**

The current study provides preliminary evidence for the role of Smart Home technology in supporting older people’s quality of life, particularly their sense of achieving in life and future security.

**Summary statement of implications for practice**

**What does this research add to existing knowledge in gerontology?**

- This study addresses the need for research on the impact of Smart Home technology to support older peoples' quality of life.
- Smart Home technology supports older peoples' overall quality of life, particularly their satisfaction with achieving in life, and sense of future security.

**What are the implications of this new knowledge for nursing care with older people?**

- Nursing care that engages SHT may improve older people's quality of life and capacity to remain independently in their own homes.

**How could the findings be used to influence policy or practice or research or education?**

- The integration of technology into the delivery of healthcare is rapidly increasing and Smart Home technology should be considered as a healthcare strategy to support older people living at home.

## 1 INTRODUCTION

Research consistently highlights a strong preference for older people to remain independent in their own homes (Kendig et al., 2017; Stones & Gullifer, 2016). The overall benefits of ageing at home include improved quality of life by supporting independence, feelings of satisfaction and fulfilment, a healthier and safer living environment, comfort pertaining to the emotional value of home, connection to community and engagement in social networks (Chen & Schulz, 2016; Kendig et al., 2017; Majumder et al., 2017). Consequently, there has been an increased interest in using innovative technology to enable Smart Homes to support people to age at home (Majumder et al., 2017), by actively or passively monitoring or mitigating the impact of health, mobility, sensory or cognitive factors on quality of life (Choi et al., 2021; Jachan et al., 2021; Majumder et al., 2017). Functional benefits of Smart Homes include supporting older people with limited mobility (e.g. remotely controlling appliances, voice activation) and memory (e.g. automated reminders to take medicine and brush teeth) (Choi et al., 2021; Jachan et al., 2021; Majumder et al., 2017; Wilson et al., 2015). Health‐related benefits of Smart Homes include health monitoring and disease management, and improved access to healthcare services (Marikyan et al., 2019; Piau et al., 2020; Wild et al., 2021; Wu et al., 2021). Smart Home technologies (SHT) such as video doorbells, light sensors and smart locks support home security and a sense of personal safety (Mamonov & Benbunan‐Fich, 2020). Carnemolla (2018) particularly identified the installation of automatic light sensors improved levels of confidence and safety for older people at risk of falling, in addition to reducing family caregiver concerns for their loved ones who live alone.

The integration of information and communication technology (ICT) into Smart Homes to support older people to maintain social connection and community engagement has also been explored (Chen & Schulz, 2016; Morris et al., 2014; Turjamaa et al., 2019). Whilst digital literacy may be a barrier for some older people to use ICT (Guner & Acarturk, 2020), the integration of intuitive communication technology such as voice interface technology has been found to improve technology adoption and acceptance (Pal et al., 2020). There is recent evidence that voice interface personal assistants such as Amazon Echo or Google Home may support older people's social health through companionship, and physical health through automated reminders, and relieve caregiver burden by offsetting daily tasks (O'Brien et al., 2020).

Whilst SHT has been found to support older people to remain independent in their own homes (Majumder et al., 2017; Ni et al., 2015; Suryadevara et al., 2013), there is little evidence of the efficacy of SHT to support older people's quality of life (Liu et al., 2016; Majumder et al., 2017), particularly in the Australian context (Turjamaa et al., 2019). Therefore, the aim of this pilot study was to explore the impact of SHT on older people's quality of life, particularly for those living alone.

The following research questions were formulated for this study.

1. Does SHT support older people's quality of life?

2. Does SHT better support quality of life for older people who live alone?

## 2 METHODS

Ethics approval was obtained from Southern Cross University Human Research Ethics Committee with Approval Number 2020/101. All participants provided informed consent.

This was a pilot study that used pre‐ and post‐intervention surveys to measure quality of life. This study adopted the widely accepted Personal Wellbeing Index (PWI; International Wellbeing Group, 2013) as the conceptual framework to measure the impact of SHT on participants subjective quality of life.

The PWI is a self‐reported measure of subjective quality of life across seven quality of life domains (standard of living, personal health, achieving in life, personal relationships, personal safety, community connectedness and future security). The PWI is a 7‐item tool measured on an 11‐point Likert scale from 0—‘no satisfaction at all’ to 10—‘completely satisfied’. The domains theoretically represent the global question ‘How satisfied are you with your life as a whole?’ (Cummins et al., 2003; International Wellbeing Group, 2013). Scores on each domain (item) are converted to a score out of 100 (e.g. a score of 7 is converted to 70). Additionally, the domain scores can be averaged to create a composite score for quality of life. The PWI has sound reliability (Cronbach's *α* = 0.88; Rodriguez‐Blazquez et al., 2011) and has been used with older people in Australia (Bennett et al., 2015; De San Miguel et al., 2017) including in the context of technology use (De San Miguel et al., 2017). The current study found the PWI to have good internal consistency (Cronbach's *α* = 0.78).

Completion of the PWI was voluntary and not a requirement of receiving the SHT.

### 2.1 The intervention

The SHT program was a 12 weeks government‐funded program, offered to clients of an aged care and disability service. The SHT program offered Google Home and assistive technology to support older people living in their own homes. The SHT program was offered free of charge, and wireless Internet data dongles were provided to participants who did not have Internet connection at home. A range of assistive technology was available including Google Home Hub, smart switches, Chromecast and Phillips Hue Smart Lighting (see Table 1 for list of assistive technology). The SHT installed in the participant's home was based on their individual goals (e.g. to become more independent, make life easier and improve home safety) and functional support needs. Participants concerned with safety and security were provided with security cameras so that they could monitor the front door or area under surveillance. Participants who had decreased mobility were provided with voice‐activated technology to operate fans, lights or the television. The layout of the SHT was also individualised to the participants’ specific home environment (e.g. whether the participants resided in a single storey or two‐storey home, and considered the homes floor plan).

**TABLE 1:** Participant characteristics (*n* = 60)

Characteristic	M (SD), range
Age	80.10 (5.56), 68–90
Gender	*n* (%)
Female	42 (70.0)
Male	18 (30.0)
Current living arrangements	
Lives alone	29 (48.3)
Does not live alone	29 (48.3)
Other	2 (3.3)
Health condition	52 (86.7)
Depression	2 (3.3)
Cancer	2 (3.3)
Diabetes	5 (8.3)
Urinary/bowel disorder	5 (8.3)
Lung disease	6 (10.0)
Chronic/regular pain	6 (10.0)
Osteoporosis	7 (11.7)
High Cholesterol	12 (20.0)
High BP	14 (23.3)
Other^a^	36 (60.0)
No health condition reported	8 (13.3)
Disability	17 (28.3)
Mobility	14 (23.3)
Vision	3 (5.0)
Cognitive^b^	2 (3.3)
Hearing	1 (1.7)
No disability reported	43 (71.7)

^a^ Other conditions included vision and hearing difficulties, heart conditions, arthritis, asthma and other respiratory and neurological conditions.

^b^ Cognitive disabilities include memory loss and vascular dementia.

Google Home allowed for automation routines, enabling several actions to be executed with one command or automatically at scheduled times. The aim of this feature was to reduce the number of direct interactions or touch points for participants with Google Home (Alam et al., 2020). For example, a participant could say ‘Goodnight Google’ and the Smart lighting would turn off including any appliances running, and an alarm set for the following day, and soothing nature noises could also be played for relaxation and deep sleep. Other examples of automation routines particularly relevant for vision‐impaired participants included turning lights on or off at certain times at night for security reasons and playing music to indicate a certain time of day. Google Home was also used to support medication and appointment times.

Technical Support Officers (TSO) skilled in the use of SHT liaised with participants to provide initial and tailored installation sessions, ongoing support and problem solving. The installation sessions lasted approximately 2–2.5 h and included a structured interview to understand each participants' individual needs. Participants were asked specific questions about their goals and needs such as *where do you spend most of your time within your house? Do you experience any functional difficulties within your home? Is there anything that you want to be able to do that you cannot currently do? What are the things that are important for you to be able to do at home that you are currently finding difficult to complete?* Upon completion of the program, participants were given the option of keeping the SHT at no cost.

### 2.2 Participant recruitment and data collection

Convenience sampling was used to recruit participants into the SHT program. Participants were recruited from an aged care and disability service in regional Australia using direct advertising of the program to their clients. To be eligible for participation in the study, participants were required to be clients of the aged care service, living in their own home, 65 years of age or over (or 45 and over if Aboriginal and Torres Strait Islander) and eligible for the Commonwealth Home Support Program (CHSP). The CHSP is a national government scheme to support older Australians who need some help to stay at home (My Aged Care, n. d.).

Prior to the commencement of the study, participants were given a study information sheet and all participants included in the study provided written consent. The survey was completed over the phone by the aged care service prior to the installation of the SHT (pre‐intervention survey) and at the conclusion of the 12 weeks program (post‐intervention survey). If a participant experienced difficulty completing the questionnaire over the phone, then a TSO attended the participants' home to complete the questionnaire in person.

### 2.3 Data analysis

All questionnaire data were manually entered into an Excel spreadsheet and then transferred into IBM SPSS Statistics (version 26) for analysis. The data were analysed descriptively to produce participant demographic data. The PWI scores were analysed pre‐ and post‐intervention using a within subject design. Differences between two‐time were compared using paired samples t‐test for parametric data or Wilcoxon signed rank tests for non‐parametric data. To detect a medium effect (*d* = 0.50) with 80% power, it was anticipated that a sample size of 52 participants was required (Brysbaert, 2019). To determine whether the magnitude of change between pre‐ and post‐program quality of life differed by living arrangement a 2 (Living arrangement: lives alone, does not live alone) x 2 (Time: pre‐program, post‐program) mixed factorial analysis of variance (ANOVA) was conducted. Living arrangement was the between groups factor, and time point was within subjects. To detect a medium effect (*d* = 0.50) in a repeated measures, F‐test with a within‐between interaction and 80% power, it was anticipated that 67 participants were required for each living arrangement (Brysbaert, 2019). Significance was considered at *p* < 0.05.

### 2.4 Assumptions

The Shapiro–Wilk test of normality indicated that the differences between pre‐ and post‐scores were only normally distributed for total PWI (*p* = 0.531), with significant deviations from normality for all PWI domains (*p* < 0.05). However, inspection of both the histograms and Q‐Q plots revealed no significant deviations from normality for quality of life or any individual domains, and therefore, the data were assumed to be approximately normally distributed (Das & Imon, 2016).

## 3 RESULTS

### 3.1 Participant characteristics

The final sample comprised 60 participants. Participant characteristics are shown in Table 2. Most participants were female and approximately half lived alone. Fifty‐two participants had one or more chronic health conditions with an average of two health conditions per participant. Seventeen participants (28.3%) reported having a disability. The most common disability was mobility followed by vision impairment. There were no differences in health issues or disabilities for older people who lived alone and those who did not live alone.

**TABLE 2:** Optional smart home technology received (*n* = 23)

Extra technology	*n* (%)
Google protect	1 (4.3)
Smoke detector	1 (4.3)
Smart lighting	6 (26.1)
Light strips	3 (13.0)
Smart vacuum	8 (34.8)
Smart mop	3 (13.0)
Smart lock	10 (43.5)
Smart doorbell	4 (17.4)
Motion sensor (turns on light)	7 (30.4)
Security camera	4 (17.4)

Whilst all participants in the SHT program received a Google Hub, Google Nest Mini, Smart switches and lighting, some clients (*n* = 23, 38.3%) also opted to receive additional SHT (Table 1). All participants who were provided with a data dongle chose to purchase their own Internet connection at the conclusion of the program and were provided with support to do so.

### 3.2 Personal wellbeing index

There were no significant differences in the PWI at baseline for gender, education, relationship status or living arrangements (all *p*s >0.528). Additionally, there was no significant correlation between age and the PWI (*p* = 0.075). Therefore, there was no cause for adjusted analysis due to differences in groups.

The PWI domains and total score are shown in Table 3. Participants' quality of life, indicated by a composite score of all domains, significantly increased after participation in the SHT program (*p* = 0.010). Participants' sense of ‘achieving in life’ (*p* = 0.026) and ‘future security’ (*p* = 0.004) domain scores were also significantly improved after participating in the SHT program. There were no statistically significant increases in ‘standard of living’, ‘personal health’, ‘personal relationships’, ‘personal safety’ or ‘community connectedness’ subscale scores.

**TABLE 3:** Pre‐ and post‐program Personal Wellbeing Index (PWI) scores *(n* = 60)

Domain	Condition	*M*	*SD*	*M* difference (post‐pre)	*p*	*d*
Total PWI (quality of life)	Pre	80.79	11.96	4.02	0.010	0.33
	Post	84.81	12.47			
Standard of living	Pre	84.67	16.62	3.50	0.051	0.23
	Post	88.17	12.95			
Personal health	Pre	74.17	20.11	4.33	0.129	0.22
	Post	78.50	18.94			
Achieving in life	Pre	77.67	17.31	5.66	0.026	0.34
	Post	83.33	16.33			
Personal relationships	Pre	84.00	19.50	1.50	0.515	0.08
	Post	85.50	19.26			
Personal safety	Pre	85.33	16.82	3.34	0.131	0.21
	Post	88.67	15.24			
Community connectedness	Pre	80.83	19.68	2.84	0.348	0.14
	Post	83.67	19.74			
Future security	Pre	78.83	17.38	7.00	0.004	0.44
	Post	85.83	14.30			

PWI scores were also compared between participants who lived alone (*n* = 29) and those who did not live alone (i.e. lived with a partner or children, *n* = 29) (Table 4). A mixed factorial ANOVA determined that improvements in quality of life and individual domain scores did not vary as a function of living arrangement.

**TABLE 4:** Pre‐ and post‐program Personal Wellbeing Index (PWI) scores by living arrangements (*n* = 58)

		Condition						
		Pre		Post				
PWI domain	Living alone	M	SD	M	SD	*M difference* (post‐pre)	*p*	η_p_^2^
Total PWI (quality of life)	Yes	78.97	10.46	82.91	11.09	3.94	0.528	0.01
	No	82.32	13.60	88.08	12.39	5.76		
Standard of living	Yes	84.14	15.00	86.55	12.61	2.41	0.450	0.01
	No	85.17	18.83	90.34	13.22	5.17		
Personal health	Yes	70.00	16.26	75.17	19.57	5.17	1.00	0.00
	No	77.59	23.40	82.76	18.11	5.17		
Achieving in life	Yes	77.59	14.06	82.07	13.73	4.48	0.454	0.01
	No	77.93	20.77	86.21	18.81	8.28		
Personal relationships	Yes	83.79	16.78	85.52	17.24	1.73	0.764	0.00
	No	84.14	22.44	87.24	20.16	3.10		
Personal safety	Yes	81.72	17.94	85.52	15.02	3.80	0.939	0.00
	No	88.97	15.66	92.41	15.27	3.44		
Community connectedness	Yes	81.72	12.84	82.41	13.54	0.69	0.152	0.02
	No	79.66	25.42	87.93	19.16	8.27		
Future security	Yes	73.79	17.61	83.10	14.66	9.31	0.598	0.01
	No	82.76	16.23	89.66	12.10	6.90		

## 4 DISCUSSION

The aim of this pilot study was to explore the impact of SHT on older people's quality of life and to explore whether the technology was particularly beneficial to those living alone. This study found that the SHT significantly supported older people's overall self‐perceived quality of life, particularly their satisfaction with achieving in life, and future security. Whilst there were no significant improvements in the standard of living, personal health, personal relationships, personal safety or community connectedness domains of quality of life, there were positive trends with small effects. There were no significant differences in quality of life scores for those who lived alone and those who did not.

A number of studies have published the health‐related and social benefits of SHT, for example, monitoring and managing health problems and improving access to healthcare services (Chen & Schulz, 2016; Marikyan et al., 2019; Morris et al., 2014; Piau et al., 2020; Turjamaa et al., 2019; Wild et al., 2021; Wu et al., 2021). However, this is one of the few studies to provide evidence of the benefits of SHT on independently living older adult's quality of life (Siegel & Dorner, 2017). Understanding the impact of SHT on older people's quality of life is important in planning and predicting the future use of home assistive technologies.

The SHT adopted by older people in this study improved their quality of life by supporting feelings of satisfaction with life and by providing a safer living environment (Chen & Schulz, 2016; Kendig et al., 2017; Majumder et al., 2017). SHT that supports older people to perform activities of daily living, particularly those activities that may have become difficult due to mobility issues or disability (e.g. gardening and cleaning), have been reported to improve satisfaction with life (Carnemolla, 2018). Personal alarms, sensor lights and remote‐control lighting have also been found to be a low‐cost option to improve a sense of security and a safer living environment (Afifi et al., 2015; Carnemolla, 2018; Corbett et al. 2021; De San Miguel et al., 2017; Tseloni et al., 2017).

Older adult's satisfaction with SHT has been found to improve when the assistive devices are personalised to their needs, supports their usual activities and is relatively easy to use (Liu et al., 2016). The automation assistive technology used in this study may have attributed to participant's quality of life by enabling their usual routine and improving experiences of independence and feeling safe. The potential role of SHT to enhance quality of life should be considered by nursing home care services to support older people to remain independent in their own homes.

Aged care policies both in Australia and globally emphasise the importance of cost‐effective innovations to support older people to remain independent in their own homes (Australian Royal Commission into Aged Care, 2021; Lui et al. 2016; World Health Organization, 2002; Siette et al., 2021). The findings of this study are particularly meaningful for application in the Australian context. Australia's healthcare system and policies for aged care support the changing needs of Australians as they age (Australian Royal Commission into Aged Care, 2021). The Commonwealth Home Support Program provides older people with access to support services to live independently and safely at home (My Aged Care, n. d.). Older people who report experiencing poor quality of life are at increased risk of admission to residential aged care (Siette et al., 2021). Therefore, the integration of SHT into home support programs should be considered a healthcare strategy to support older people's quality of life and ability remain independent at home.

There were no significant differences in quality of life scores for those who lived alone and those who did not. Previous research has reported that older peoples' living arrangements have minimal impact on their perceived quality of life, rather socio‐economic factors are more important in developed countries like Australia (Lim & Kua 2011; Yahaya et al., 2010). Social cohesion has been found to buffer the negative impacts of living alone on quality of life (Huang et al., 2020). Further research is needed to better understand the impact of SHT and living arrangements on older people's quality of life.

## 5 CONCLUSION

This pilot study found that SHT may support older people's quality of life, particularly in the domains of achieving in life and future security. However, the generalisability of the findings is limited by the small sample size (*n* = 60) and the impact of the COVID‐19 pandemic on recruitment. Regardless, these findings make a unique contribution to the literature as there is limited empirical evidence for the capacity of SHT to support older people's quality of life (Liu et al., 2016; Majumder et al., 2017). As cost‐effective healthcare strategies are sought to support older people to live independently at home (Tun et al., 2021), the findings of this research may inform government policy and aged care services, including the CHSP (My Aged Care, n. d.) to consider SHT to support older people quality of life and ability remain independent at home.

## CONFLICT OF INTEREST

The authors are not aware of any conflict of interest.

## Data Availability

Data availability subject to third party restrictions.
